# Clozapine and COVID-19

**DOI:** 10.1192/bjb.2020.66

**Published:** 2020-08

**Authors:** Edward Silva, Siobhan Gee, Shubulade Smith, Fiona Gaughran

**Affiliations:** Consultant Psychiatrist, Ashworth Hospital, Mersey Care NHS Foundation Trust, UK; email: ed.silva@merseycare.nhs.uk; Principal Pharmacist, South London and Maudsley NHS Foundation Trust, Maudsley Hospital, UK; Consultant Psychiatrist, South London and the Maudsley NHS Foundation Trust and Visiting Senior Lecturer, Institute of Psychiatry, Psychology and Neuroscience, King's College London, UK; Consultant Psychiatrist, National Psychosis Unit, South London and the Maudsley NHS Foundation Trust and Reader, Institute of Psychiatry, Psychology and Neuroscience, King's College London, UK

The COVID-19 pandemic presents psychiatrists prescribing clozapine with complexities over and above the general difficulties already described among Chinese psychiatric in-patients.^1^ Some common initiation effects of clozapine: isolated fever, tachycardia or a mildly raised C-reactive protein^2^ may be difficult to differentiate from intercurrent infection, including COVID-19.^3^ Other laboratory anomalies may add to confusion. Given COVID-19 infection deaths that are not the result of acute respiratory distress syndrome are often from fulminant myocarditis, for which a raised troponin is a poor prognostic feature, troponin levels taken routinely during clozapine initiation will need to be interpreted carefully. However, while leucopenia is a reported blood dyscrasia with COVID-19, to date neither neutropenia nor agranulocytosis is.

In the absence of an antibody test for COVID-19^4^ we rely on reverse-transcriptase polymerase chain reaction COVID-19 testing, which does not have 100% sensitivity in the initial phase of infection, so a single negative test does not exclude infection. Once an antibody test becomes available it will be a useful addition to pre-clozapine investigations.

Comorbidities such as diabetes, hypertension, respiratory illness and cardiovascular disease are very common in patients taking clozapine but are associated with adverse outcomes in the event of COVID-19 infection, including increased mortality rates. It is not known whether the antibody deficiency described in patients taking clozapine will further compromise this vulnerable group.^5^ These are not, however, indications to stop clozapine, which itself has serious adverse consequences. Rather patients need clear advice and, if possible, assistance regarding self-isolation and other precautions advised. Obesity and sleep apnoea may also contribute to poor outcomes and continuous positive airway pressure treatment, which can be an aerosol-generating procedure,^6^ may be a risk to staff.

Individuals already established on clozapine and managed in the community may require changes to their management. In view of recommendations for social distancing, the use of clozapine clinics for routine blood testing should be reconsidered. Instead, blood tests may be better performed in patient's homes, with staff using personal protective equipment and at, or near, the maximum intervals permitted. While the standard blood monitoring frequencies are at weekly (weeks 1–18), fortnightly (weeks 19–52) and four-weekly (over 52 weeks) intervals, clozapine can still be dispensed and administered with satisfactory monitoring at 14-, 21- and 42-day intervals, respectively.

Inevitably many patients taking clozapine will present with flu-like symptoms. An urgent full blood count will be required to exclude neutropenia with appropriate action. Many, however, will have another cause and so the evolving National Health Service recommendations regarding isolation and hopefully testing for COVID-19 infection should be followed. The combination of flu-like symptoms, chest pain and shortness of breath will, as community prevalence of COVID-19 increases, be much more likely to be because of COVID-19 than clozapine-induced myocarditis, except perhaps within the first 60 days of treatment.^7^ However, such a presentation will still need investigation and cessation of clozapine may on occasion be required as well as urgent general medical assistance. Careful documentation of symptom profiles and investigations will aid subsequent decisions regarding clozapine re-challenge. In the event of COVID-19 infection the acute-phase reaction may result in reduced activity of cytochrome P450 1A2, raising clozapine levels and so an urgent trough clozapine level will be needed with a reduction in clozapine dose if required. This effect may be amplified if hospital admission is indicated necessitating abrupt change in smoking habits.

In summary; COVID-19 presents us with extreme difficulties regards clozapine initiation, the risks of COVID-19 are insufficient to justify stopping clozapine, an action which presents its own serious problems, precautions must be taken now to help protect our high-risk patients as COVID-19 infection may jeopardise both their physical and mental health.
